# Case Report: Dental management of an 8-year-old child with alternating hemiplegia of childhood under general anesthesia: clinical challenges and multidisciplinary considerations

**DOI:** 10.3389/fdmed.2026.1940425

**Published:** 2026-10-14

**Authors:** Asha B. Thomas, Sandra Maria Enchackal, Shailesh Shenoy, Manjula Sudhakar Rao, Sundeep Hegde K.

**Affiliations:** 1 Department of Pediatric and Preventive Dentistry, Yenepoya Dental College, Yenepoya (Deemed to be University), Mangalore, India; 2 Department of Anaesthesia, Yenepoya Medical College, Yenepoya (Deemed to be University), Mangalore, India

**Keywords:** alternating hemiplegia of childhood, ATP1A3, dental rehabilitation, general anesthesia, neurodevelopmental disorders, pediatric dentistry, special child

## Abstract

**Background:**

Alternating Hemiplegia of Childhood (AHC) is an ultra-rare neurodevelopmental disorder caused by pathogenic *ATP1A3* variants, characterized by recurrent hemiplegic episodes, dystonia, autonomic dysfunction, and intellectual disability. The dental environment represents a recognized trigger for paroxysmal episodes, rendering routine outpatient care unfeasible in affected children.

**Case description:**

An 8-year-old male with a confirmed *de novo ATP1A3* variant presented with extensive dental disease including a grossly decayed tooth 26, multiple carious lesions, retained root stumps, and generalized poor oral hygiene. Comprehensive dental rehabilitation was performed under general anesthesia following multidisciplinary fitness clearance.

**Clinical management and outcome:**

Total intravenous anesthesia with propofol and dexmedetomidine was employed, with deliberate avoidance of succinylcholine and volatile agents; rocuronium and prophylactic levetiracetam were used perioperatively. Extractions, restorations, and scaling were completed without intraoperative or postoperative hemiplegic, seizure, or autonomic events. The patient recovered uneventfully following 24-hour PICU observation.

**Conclusions:**

In this child with genetically confirmed AHC, an individualised, multidisciplinary total-intravenous-anaesthesia protocol allowed comprehensive dental rehabilitation to be completed without hemiplegic, seizure, or autonomic complications. This single case suggests that such a protocol can be safe in AHC, although the disease burden observed underscores the need for earlier preventive dental engagement and caregiver education in this population.

## 1 Introduction

Alternating Hemiplegia of Childhood (AHC) is a rare and complex neurodevelopmental disorder with an estimated prevalence of 1 in 1,000,000 individuals and an annual incidence of less than 1 in 100,000 newborns (1). First described by Verret and Steele in the late 1960s in a cohort of eight children with migraine, among whom three demonstrated the characteristic features of the condition (2), AHC was subsequently delineated as a distinct clinical entity through diagnostic criteria established by Krageloh and Aicardi (1) and Bourgeois et al. (3). These criteria include: onset before 18 months of age; repeated episodes of hemiplegia involving one or both sides of the body; autonomic phenomena; cognitive impairment; neurological abnormalities such as choreoathetosis; and a characteristic resolution of symptoms during sleep with resumption upon waking (1, 3).

The clinical presentation of AHC is heterogeneous and follows a unique sequential pattern. Paroxysmal manifestations include hemiplegic episodes of variable frequency and duration lasting from minutes to days, dystonic seizures, epilepsy, oculomotor abnormalities, and autonomic dysfunction, often precipitated by environmental stressors such as temperature changes, emotional stress, or bathing (4). Non-paroxysmal features include developmental delay, intellectual disability, movement disorders, ataxia, dystonia, choreoathetosis, and abnormal eye involvement (5).

The patient reported symptom exacerbation following physical activity and sunlight exposure. In the literature, other neurologic comorbidities associated with the condition include ataxia, choreoathetosis, and cognitive impairment. Reported triggering events and environmental influences have been broadly categorized into five groups: (1) stress or physical activity; (2) specific foods; (3) light exposure; (4) exposure to water, such as swimming or bathing; and (5) medications (6). In the present case, physical activity and sunlight exposure were identified as specific triggers of the symptoms.

The molecular underpinning of AHC has been substantially clarified over the past two decades. Pathogenic mutations in the *ATP1A3* gene, encoding the *α*3 subunit of the Na⁺/K⁺-ATPase transmembrane ion pump, represent the predominant genetic etiology, with mutations in *ATP1A2* accounting for a smaller proportion of cases (7). Functionally, reduced *α*3 subunit activity impairs the maintenance of physiological sodium and potassium gradients across neuronal membranes, thereby disrupting neurotransmitter reuptake including that of glutamate and *γ*-aminobutyric acid (GABA) and promoting abnormal neuronal excitability, which is hypothesized to underlie the paroxysmal manifestations of AHC (4).

From a dental management perspective, children with AHC present unique and significant challenges. The disorder's multisystemic nature encompassing intellectual disability, behavioral unpredictability, complex pharmacological regimens, and susceptibility to environmentally triggered paroxysmal episodes frequently renders conventional outpatient dental treatment unfeasible (4). The dental environment itself, with its inherent stimuli including procedural stress, pain, anxiety, and temperature fluctuations, represents a recognized precipitant of hemiplegic episodes (4). General anesthesia (GA) therefore emerges as a preferred and often necessary modality for comprehensive dental rehabilitation in this population, facilitating safe and atraumatic care while minimizing triggers for paroxysmal events.

We present the case of an 8-year-old child with a confirmed diagnosis of AHC, harbouring a *de novo* pathogenic variant in *ATP1A3*, who underwent successful comprehensive dental rehabilitation under general anesthesia, with the aim of highlighting the perioperative and clinical considerations relevant to the dental management of this condition within special care dentistry.

## 2 Case report

### 2.1 Patient information

An 8-year-old male patient, weighing 16 kg, was referred to the Department of Pediatric and Preventive Dentistry with a chief complaint of pain and swelling in the upper left posterior tooth region of approximately one month’s duration.

According to his mother, who served as the primary caregiver, the onset of dental pain had been insidious, gradually worsening over the preceding four weeks and was accompanied by visible facial swelling in the left upper posterior region. Upon noticing the swelling, the mother had initially sought consultation at a private dental clinic, where the patient was prescribed medication including analgesics and antibiotics for the management of the swelling and associated pain.

The mother further reported a notable behavioral indicator of dental pain, the patient would habitually chew on his clothing during episodes of discomfort, a self-stimulatory response attributed to his inability to verbally communicate the severity or precise location of pain. No systemic symptoms such as fever or lymphadenopathy were reported at the time of presentation to our department.

### 2.2 Medical history

The patient was born of a non-consanguineous marriage with no significant perinatal complications reported. He presented with clinical features of motor developmental delay, central hypotonia, and periodic paralysis of limbs from as early as 5 months of age. Brain MRI and electroencephalogram (EEG) performed during the initial diagnostic workup were reported as within normal limits. Based on the clinical presentation, a provisional diagnosis of Alternating Hemiplegia of Childhood was suspected, with differential considerations including channelopathies and mitochondrial disorders (8). The patient had been under regular neurological follow-up since the time of diagnosis.

### 2.3 Clinical findings

On extraoral examination, the facial swelling had completely resolved following the course of antibiotics and analgesics prescribed at the private clinic, with no residual asymmetry.

Caries status was recorded using the International Caries Detection and Assessment System (ICDAS-II). Tooth 26 presented with an ICDAS code 6 (extensive distinct cavity with visible dentine). Teeth 16, 36, and 46 demonstrated ICDAS code 5 (distinct cavity with visible dentine) without clinical signs of pulpal exposure. Tooth 22 presented with ICDAS code 3 (localized enamel breakdown with no visible dentine).

The overall dental findings were documented as follows:

- Tooth 26: Grossly decayed with extensive crown destruction and suspected pulpal involvement, consistent with the chief complaint of pain and localized swelling in the upper left posterior region.
- Teeth 16, 36, and 46: Dentinal caries requiring operative restorative intervention.
- Teeth 53, 54, and 55: Retained root stumps with no clinical evidence of associated soft tissue pathology at the time of examination.
- Tooth 22: Smooth surface caries noted on the labial aspect.
- Oral Hygiene Assessment: Oral hygiene was quantitatively assessed using the Simplified Oral Hygiene Index (OHI-S), which recorded a score of 3.2 (interpreted as poor oral hygiene) (9). This corresponded to generalized supragingival calculus and heavy plaque deposits observed across multiple sextants, indicative of long-standing inadequate oral hygiene maintenance. Standardized scoring was considered particularly relevant given the well-documented higher caries burden in children with neurodevelopmental impairment.

### 2.4 Therapeutic intervention

Preoperative workup involving full blood count, serology, chest x-ray, EEG, and brain MRI/ MRA revealed no contraindication to anesthesia, which was considered safe for mild to moderate anesthetic risk. With multidisciplinary clearance and consent from parents, full mouth rehabilitation was performed under general anesthesia with endotracheal intubation uneventfully. Intraoperatively, ECG, pulse oximetry, capnography, and non-invasive blood pressure measurement were monitored. Extraction of gross decay and non-restorable tooth 26 with associated dentoalveolar infection; (Figure 1) extraction of retained roots of teeth 53, 54, and 55. Teeth 16, 36, and 46 presented with deep dentinal caries without clinical or radiographic evidence of irreversible pulpal or periapical involvement; therefore, indirect pulp therapy was performed with the aim of preserving pulp vitality. Following selective caries removal (10), Biodentine® (Septodont, Saint-Maur-des-Fossés, France), a calcium silicate-based bioactive dentin substitute, was placed over the remaining affected dentin. The teeth were subsequently restored with EQUIA Forte® (GC Corporation, Tokyo, Japan). Tooth 22 was restored with a glass hybrid restorative system (GC EQUIA Forte HT, GC Corporation, Tokyo, Japan). This reinforced material was selected over conventional glass ionomer cement due to its superior compressive strength, flexural strength, and wear resistance, which permitted rapid single-increment bulk placement within the strict time constraints of general anesthesia while providing long-term fluoride release and chemical adhesion. Supragingival scaling and oral prophylaxis were performed intraoperatively. There were no intraoperative complications such as hemiplegia, seizure, and autonomic dysfunction. Postoperatively, the patient was transferred to the pediatric intensive care unit where he was observed for adequacy of breathing, hemodynamic stability, maintenance of body temperature, and neurological function as well as signs of dystonia, hemiplegia, and malignant hyperthermia. Analgesia was achieved with intravenous paracetamol to minimize opioid-related respiratory depression, and a calm, low-stimulation environment was maintained to reduce the risk of stress-induced neurological exacerbations. Normothermia and adequate hydration were ensured throughout. No postoperative neurological events or adverse anesthetic sequelae were observed; the patient was stepped down to the ward at 24 h and discharged following satisfactory recovery (Figure 2). Caregiver-directed oral hygiene instructions and dietary guidance were provided at discharge, with emphasis on assisted oral hygiene maintenance in view of the patient's neurodevelopmental limitations. The caregiver was instructed to use a standard 1,450 ppm fluoridated toothpaste for twice-daily brushing. At the one-month postoperative follow-up visit, the patient demonstrated uneventful healing and stable restorations. During this recall visit, a topical fluoride application (MI Varnish, GC Corporation, Tokyo, Japan) was applied across the dentition to aid remineralization and prevent secondary lesion development. A structured preventive recall protocol has been established to monitor his oral health, restoration integrity, and preventive care compliance at 3-month intervals up to one year.

**Figure 1:**
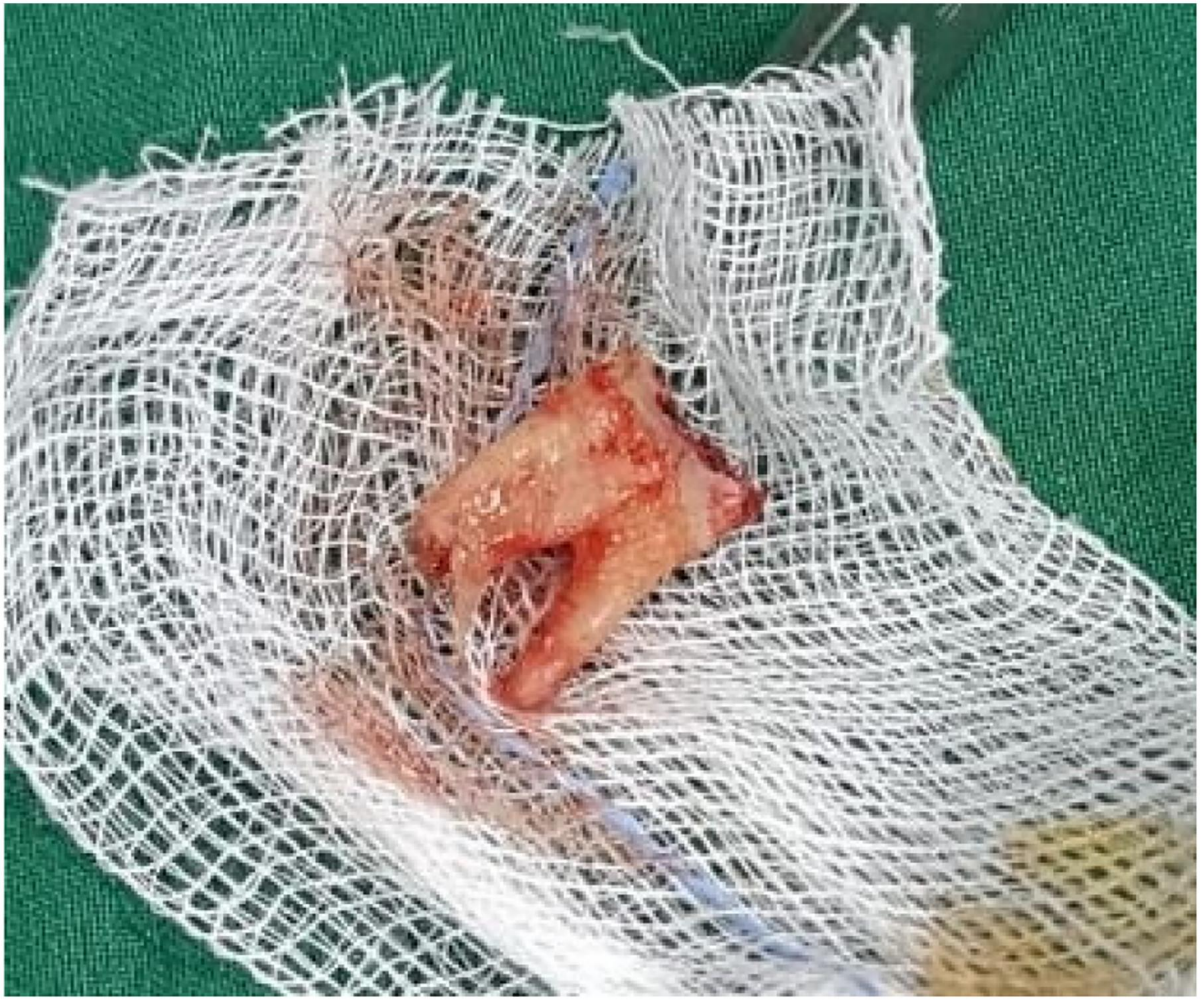
Extracted tooth in relation to 26. Captured by author in Samsung S 24 Ultra.

**Figure 2:**
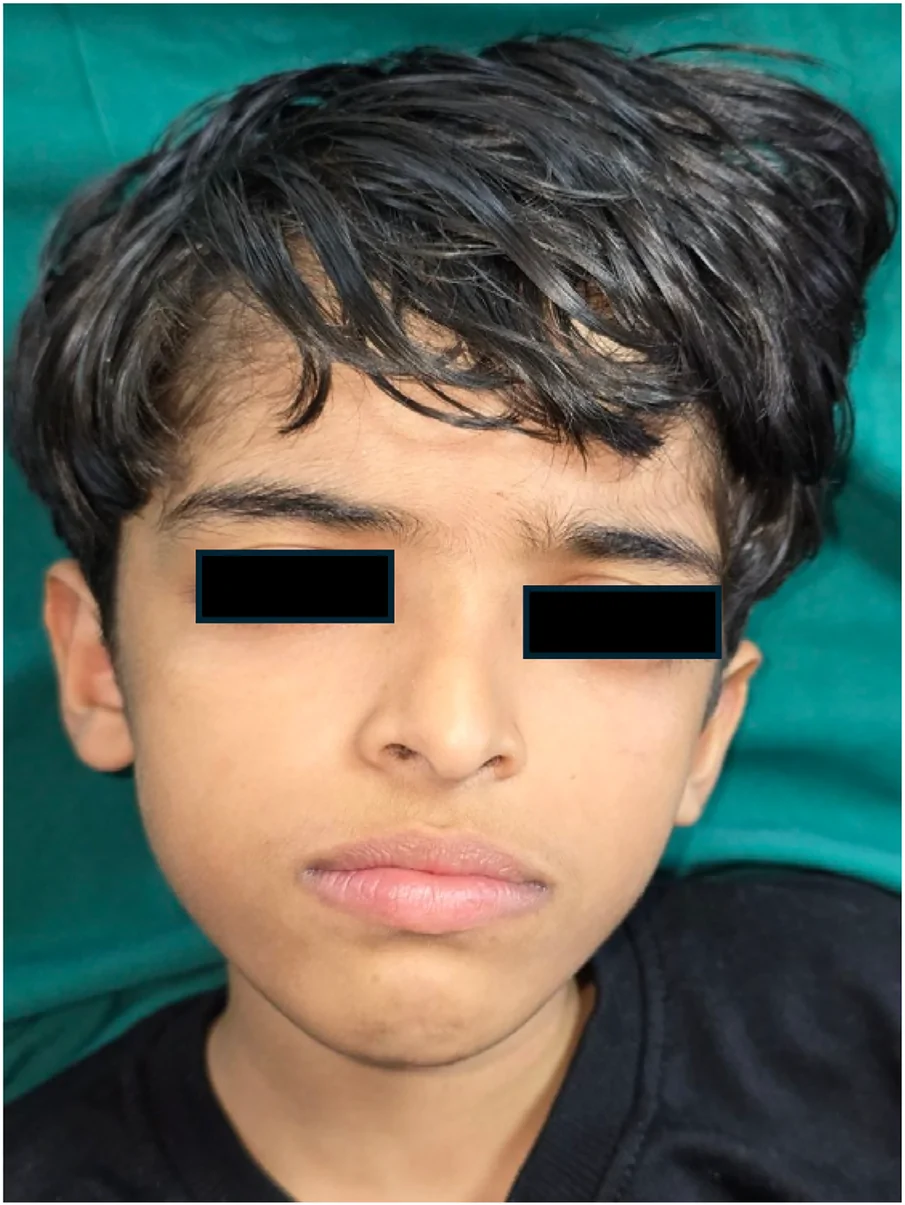
Postoperative extraoral photograph. Captured by author in Samsung S 24 Ultra.

The patient’s clinical information and therapeutic aspects were summarized in Table 1.

**Table 1:** Clinical summary of the reported case key clinical and therapeutic aspects.

Category	Details
Demographic data	8-year-old male
Weight	16 kg
Chief complaint	Pain and swelling in the upper left posterior tooth region since 1 month
Medical history	Known case of Alternating Hemiplegia of Childhood (AHC); confirmed *de novo* heterozygous pathogenic variant in *ATP1A3,* motor developmental delay and central hypotonia since 5 months of age
Current medications	Tablet Topiramate 25 mg (1-0-1); Tablet Sibelium (flunarizine) 10 mg (1-0-1); Syrup Rexite (cinnarizine) 2.5 mL (morning); Deksel Neo 60,000 IU (weekly × 4 weeks, then monthly × 6 months)
Associated conditions	Intellectual disability; neurodevelopmental delay; limited verbal communication; vitamin D deficiency
Preoperative investigations	Complete blood count (CBC); serological tests; chest radiograph; electroencephalogram (EEG); MRI with MRA of brain
Anesthetic risk	Mild-to-moderate risk
Anesthetic precautions	Levetiracetam (Levipil®) 30 mg/kg IV for seizure management; succinylcholine avoided — rocuronium preferred for rapid sequence intubation; propofol via target-controlled infusion (TCI); volatile inhalational agents used with caution
Affected teeth	Grossly decayed tooth 26; dentinal caries wrt 16, 36, 46; root stumps wrt 53, 54, 55; smooth surface caries wrt 22; generalized supragingival calculus
Diagnosis	Severe early childhood caries with dentoalveolar infection (tooth 26); multiple carious lesions; retained root stumps; poor oral hygiene in a medically compromised child with AHC
Treatment performed under GA	Extraction of grossly decayed tooth 26; extraction of root stumps (53, 54, 55); restoration of dentinal caries (16, 36, 46); management of smooth surface caries (22); supragingival scaling and oral prophylaxis
Postoperative medications	Injection Taxim-O (cefixime) 500 mg IV; Injection pantoprazole 20 mg IV; Injection paracetamol 250 mg IV
Outcomes	Uneventful recovery from general anesthesia; no intraoperative or postoperative neurological episodes; hemodynamic stability maintained throughout; successful comprehensive dental rehabilitation
Preventive measures	Caregiver oral hygiene education; dietary counselling; regular dental follow-up recommended; caregiver-assisted tooth brushing advised
Timeline	Day 1: Presentation (pain) → Day 2: Preop workup & medical clearance → Day 3: Consent & GA dental rehabilitation → Day 4: PICU monitoring & discharge → 1-Month Recall: Healing confirmed & topical MI Varnish application

### 2.5 Patient/caregiver perspective

As the primary historian, the patient's mother identified clothing-chewing as his main indicator of dental pain and participated closely in treatment decisions. Though initially anxious about general anesthesia given his complex neurological background, she noted immediate pain relief, cessation of clothing-chewing, and improved eating comfort postoperatively. At the one-month recall, she reported complete satisfaction with his recovery, enhanced daily comfort, and easier oral hygiene maintenance.

## 3 Discussion

Alternating Hemiplegia of Childhood (AHC) is a rare neurodevelopmental disorder characterised by recurrent episodes of hemiplegia, dystonia, autonomic dysfunction, and abnormal ocular movements. The extent of dental disease observed in this patient including grossly decayed tooth 26, dentinal caries involving multiple permanent and primary teeth, retained root stumps, and generalized poor oral hygiene is consistent with the well-documented predisposition to poor oral health in children with neurodevelopmental disorders and intellectual disability.

The decision to perform dental rehabilitation under general anesthesia in this patient was guided by the patient's inability to cooperate for treatment in the outpatient dental setting. In pediatric patients with AHC, dental treatment presents a dual challenge: addressing an urgent dental disease burden while avoiding triggers that can precipitate catastrophic neurological or cardiovascular deterioration. The perioperative anesthetic management of this patient with AHC presented several unique and potentially life-threatening challenges. In a comprehensive cohort study evaluating sedation and anesthetic outcomes in AHC patients, Parker et al. (2022) reported a high rate of perioperative complications, particularly cardiac arrhythmias, prolonged emergence, respiratory depression, and stress-induced paroxysmal motor spells (11). The most critical concern was the risk of stress-induced paroxysmal hemiplegic or dystonic episodes, given the well-established sensitivity of *ATP1A3*-related disorders to physiological stressors inherent to the perioperative environment, including pain, anxiety, and temperature fluctuations (4).

Total intravenous anesthesia with propofol and dexmedetomidine was employed, avoiding volatile agents and succinylcholine to mitigate risks of neurological deterioration and malignant hyperthermia, while dexmedetomidine provided additional sympatholytic, sedation, and opioid-sparing analgesia with preserved respiratory function (12). This anesthetic selection directly aligns with international consensus guidelines for AHC, which advocate for Total Intravenous Anesthesia (TIVA) to prevent autonomic hyper-reactivity and avoid volatile agents that can cause post-anesthetic agitation (OrphanAnesthesia, 2021) (13). The theoretical risk of malignant hyperthermia susceptibility in the context of underlying membrane channelopathy, while not definitively established for AHC, warranted precautionary avoidance of succinylcholine, with rocuronium designated as the neuromuscular blocking agent of choice. Furthermore, given that approximately 50% of AHC patients experience comorbid epilepsy and up to 25% experience refractory status epilepticus, as reported by Samanta et al. (14), Because epilepsy is an important neurological comorbidity in AHC, perioperative seizure risk was also considered in the anaesthetic plan. Levetiracetam 30 mg/kg IV was administered following multidisciplinary neurological assessment as antiseizure prophylaxis (15). Importantly, its use was directed toward seizure-risk management and was not intended as disease-specific prophylaxis against AHC-related hemiplegic attacks, all of which collectively contributed to the uneventful perioperative course observed in this case.

The caries burden observed in this patient a non-restorable first permanent molar, dentinal caries in three further permanent molars, retained infected primary roots, and generalized calculus mirrors findings reported in other neurodevelopmentally impaired populations. Using ICDAS-II criteria comparable to those applied in the present case, Pryimak et al. found significantly greater caries severity and a higher frequency of deep cavitated lesions in children with cerebral palsy as motor impairment worsened, attributing this to caregiver-dependent hygiene and reduced access to preventive care factors equally applicable to AHC (16). Similarly, in a long-term outcome study of children treated under GA, Liatsi et al. found that special-needs patients had significantly higher restored and decayed surface scores on long-term follow-up than healthy children treated under the same modality, reinforcing that the underlying neurodevelopmental vulnerability, rather than the GA episode itself, drives the disproportionate disease burden and recurrence risk in this population (17). These findings support our contention that AHC should be regarded as a high caries-risk condition requiring dental integration into neurological follow-up rather than reactive, crisis-driven GA-based care.

Following multidisciplinary fitness clearance and parental informed consent, the patient underwent comprehensive dental rehabilitation under general anesthesia with uneventful endotracheal intubation and continuous intraoperative monitoring. Treatment planning in this case prioritised procedures that would eliminate active infection and arrest disease progression within a single, non-repeatable anaesthetic exposure, given the substantial anaesthetic risk profile of AHC. Extraction was reserved for the non-restorable tooth 26 and the infected retained roots 53–55, Indirect pulp therapy was preferred for teeth 16, 36, and 46 as a conservative vital pulp therapy approach for deep caries without evidence of irreversible pulpal disease, aiming to preserve pulp vitality. Biodentine®, a calcium silicate-based bioactive material, was used to support pulpal healing (10), followed by definitive restoration with EQUIA Forte® to achieve an effective coronal seal (18). The restorative material for tooth 22 was selected to balance moisture tolerance against the mechanical demands of a stress-bearing restoration placed without the possibility of a same-day cooperative recall. This decision-making sequence favouring the most conservative option compatible with long-term success at each site is consistent with recommendations for GA-based comprehensive care in special-needs children, where the number of anaesthetic exposures over a child's lifetime should itself be treated as a risk to be minimised (19). Preventive measures instituted at discharge, together with the recall protocol described above, aimed to extend the benefit of this single GA episode for as long as possible, reflecting evidence that structured recall rather than the GA procedure itself is the principal determinant of long-term oral health in this population (17).

As demonstrated in this case, children with AHC present with significant dental disease burden compounded by their underlying neurodevelopmental limitations, caregiver-dependent oral hygiene, and restricted access to specialized dental services. Increasing awareness of AHC among dental professionals will enable timely referral, early preventive intervention, and appropriate multidisciplinary care planning. Early dental visits should be initiated concurrently with neurological follow-up, focusing on caregiver oral hygiene education, dietary counselling, and regular fluoride prophylaxis to reduce caries risk (20). Dental treatment under local anesthesia remains the ideal modality where cooperation permits; however, when behavioral limitations and neurodevelopmental complexity preclude outpatient management, general anesthesia with individualized perioperative protocols remains the safest and most effective option for comprehensive dental rehabilitation (21). Enhanced preventive care, earlier dental recall intervals, and proactive multidisciplinary collaboration are essential to minimize disease burden and avoid the need for repeated general anesthesia in this vulnerable patient population.

This report is subject to the inherent limitations of single-case design: findings cannot be generalised to the wider AHC population, whose phenotypic and anaesthetic-risk heterogeneity is well recognised, and the one-month follow-up reported here is insufficient to draw conclusions about long-term restoration survival or caries recurrence. Comparative cohort data or a case series across multiple AHC patients would be needed to validate the anaesthetic and dental protocols described here as a generalisable standard of care.

## 4 Conclusion

This case report demonstrates that comprehensive dental rehabilitation was safely accomplished in an 8-year-old child with genetically confirmed Alternating Hemiplegia of Childhood (AHC) using a tailored, multidisciplinary approach and a total intravenous anesthesia (TIVA) protocol. Throughout the perioperative period, no paroxysmal hemiplegic spells, seizure activity, or autonomic instability occurred, validating the safety of the specific protocol employed in this instance. While findings from a single case cannot be generalized to all patients with AHC, the successful management of this patient underscores the value of proactive coordination between pediatric dentists, anaesthesiologists, and neurologists when treating children with complex channelopathies. Establishing early preventive care and caregiver oral hygiene guidance remains critical to maintaining oral health and minimizing the need for future general anesthesia exposures in this patient.

## Data Availability

The original contributions presented in the study are included in the article/supplementary material, further inquiries can be directed to the corresponding author/s.
